# Novel Immunity Proteins Associated with Colicin M-like Bacteriocins Exhibit Promiscuous Protection in *Pseudomonas*

**DOI:** 10.3389/fmicb.2017.00093

**Published:** 2017-01-30

**Authors:** Maarten G. K. Ghequire, Lieselore Kemland, René De Mot

**Affiliations:** ^1^Centre of Microbial and Plant Genetics, KU LeuvenHeverlee, Belgium; ^2^National Institute of Diabetes and Digestive and Kidney Diseases, National Institutes of HealthBethesda, MD, USA

**Keywords:** colicin M, PmiA, PmiB, pyocin, peptidoglycan, lipid II

## Abstract

Bacteriocins related to colicin M, acting via cleavage of the cell wall precursor lipid II, have been characterized in γ- and β-proteobacteria. Depending on the species, immunity is provided by either an inner membrane-anchored periplasmic protein or by an integral membrane protein. In *Pseudomonas* however, the immunity partner of colicin M-like bacteriocins remains unknown. Based on an *in silico* analysis in pseudomonad genomes, we here identify a gene encoding a putative immunity partner that represents a novel type of integral membrane protein (PmiA, *Pseudomonas* colicin M-like immunity type A). By heterologous expression of *pmiA* genes in susceptible strains, we show that immunity to colicin M-like bacteriocins is indeed provided by the cognate PmiA. Sequence homology among PmiA proteins is essentially absent, except for a short motif with a conserved periplasm-exposed aspartate residue. However, PmiA's protective function is not abolished by changing this acidic residue to the uncharged alanine. Immunity by PmiAs appears promiscuous to the extent that PmiA homologs from a clade sharing <40% pairwise amino acid identity, equally provide protection against the bacteriocin linked to the original PmiA. This study shows that multiple immunity factors have evolved independently to silence lipid II-targeting enzymatic bacteriocins. Their relaxed bacteriocin immunization capacity contrasts to the strict specificity of immunity proteins shielding the enzymatic domain of nuclease bacteriocins. The nature of associated immune functions needs consideration when using such natural protein antibiotics or designing novel variants.

## Introduction

Antibacterial proteins are currently subject of renewed interest in the search for antimicrobials with novel modes of action. As yet poorly explored potential sources of such alternative protein-based antibiotics include lytic bacteriophages (Gerstmans et al., 2016) and bacteriocinogenic strains (Schulz et al., 2015). Bacteriocins are key mediators of intra-species competition between bacteria and combine high specificity with single-hit killing potency. Prominent bacteriocin producers that target human and plant pathogenic Gram-negative bacteria are found among enterobacteria (Cascales et al., 2007; Holt et al., 2013; Grinter et al., 2014; Schulz et al., 2015) and pseudomonads (Ghequire and De Mot, 2014).

*Pseudomonas* bacteriocins are very diverse with respect to structure and mode of action. R-type and F-type tailocins are multiprotein complexes that are evolutionary related to bacteriophage tails (Ghequire and De Mot, 2015a; Ghequire et al., 2015; Hockett et al., 2015). Lectin-like bacteriocins are constituted of a tandem of monocot mannose-binding lectin domains, and kill target cells via an unknown mechanism (Ghequire et al., 2013, 2014; McCaughey et al., 2014). S-type bacteriocins are multi-domain toxins with a modular structure similar to colicins, the bacteriocins from *Escherichia coli* (Cascales et al., 2007; Ghequire and De Mot, 2014; Dingemans et al., 2016; McCaughey et al., 2016). Self-inhibition of *Pseudomonas aeruginosa* strains producing a S-type bacteriocin (S pyocin) is circumvented by an immunity protein that transiently impedes the bacteriocin's toxic function. This immunity complement is co-expressed with the bacteriocin, and is typically encoded downstream of the pyocin gene, on the same or opposite strand. In the case of S-type bacteriocins with nuclease activity, bacteriocins are secreted as high-affinity bacteriocin-immunity protein complexes (Joshi et al., 2015). *Pseudomonas* colicin M-like bacteriocins (PseuMs) host a lipid II-cleaving domain, homologous to the catalytic domain of colicin M (ColM; Barreteau et al., 2009, 2012; Grinter et al., 2012b). However, in contrast to S pyocin-immunity complexes, it is not clear how PseuM producers protect themselves. Inspection of their *pseuM* genomic context did not reveal homologs of *cmi*, the cognate immunity partner for ColM in *E. coli*, nor were such homologs detected elsewhere in their genomes (Barreteau et al., 2009).

In *E. coli*, the *cmi* gene is located downstream of the bacteriocin gene on the opposite strand (Olschläger et al., 1984; Olschläger and Braun, 1987). Located in the periplasm, Cmi is anchored to the inner membrane by an amino-terminal hydrophobic α-helix, assisting in secretion but not being cleaved off. There, Cmi transiently impedes colicin M's activity during secretion and inactivates imported ColM (Olschläger and Braun, 1987; Olschläger et al., 1991; Gross and Braun, 1996). The crystal structure of Cmi revealed the presence of four α-helices and four β-strands, and is exemplary of a YebF fold (Gérard et al., 2011; Usón et al., 2012). In *Pectobacterium* a *cmi*-like gene is located downstream and opposite of the pectocin M gene, but its protective functionality remains to be demonstrated (Grinter et al., 2012a). In most strains of *Burkholderia* producing colicin M-like burkhocins, immunity is mediated by *bmiB* genes that give rise to proteins with a small size and predicted topology similar to Cmi (Ghequire and De Mot, 2015b). However, the amino acid sequence of the periplasmic moiety of BmiB is unrelated to Cmi and lacks the characteristic YebF domain. Fewer strains carry a second type of burkhocin immunity gene (*bmiA*) that encodes a small integral membrane protein comprising three transmembrane helices.

In this study we investigated the genomic context of *pseuM* genes, in search for candidate immunity partners. We demonstrate that a gene downstream of *pseuM* provides cognate immunity. Apart from sharing a common membrane topology reminiscent of BmiA proteins, the encoded proteins display very poor sequence conservation across PseuM-bacteriocinogenic pseudomonads. The possible role of a short semi-conserved motif in these proteins is explored, as well as the capability of PmiA proteins to immunize targeted cells in an (a)specific way.

## Materials and methods

### Genome searches and phylogenetic analysis

*PseuM* bacteriocin genes in *Pseudomonas* genomes were identified by homology searches using the National Center for Biotechnology (NCBI) non-redundant database. The Pfam Colicin_M module PF14859 was used as a query. Genes downstream of unique *pseuM* bacteriocin gene products were selected for further analysis. Multiple sequence alignments were generated with MUSCLE and phylogenetic reconstructions were carried out with PhyML (1000 bootstrap replicates), implemented in Geneious v7.1.7. The JTT substitution model (Jones et al., 1992) was used for studying the evolution of a large number of amino acid sequences. Amino-terminal signal sequences were predicted by PRED-TAT (http://www.compgen.org/tools/PRED-TAT) and TOPCONS (http://topcons.cbr.su.se/). Transmembrane regions and topology were predicted by TMHMM (http://www.cbs.dtu.dk/services/TMHMM/), and TOPCONS.

### Bacterial strains and media

Strains used in this study are listed in Table S1. *E. coli* was grown in LB (MP Biomedicals) at 37°C, whereas *Pseudomonas* strains were grown with shaking at 200 rpm in Trypticase Soy Broth (TSB, BD Biosciences) and Casamino Acids Medium (CAA, BD Biosciences) at 30°C, except for *P. aeruginosa* that was grown at 37°C. Growth media were supplemented with agar (1.5%, Invitrogen), filter-sterilized isopropyl-β-D-thiogalactopyranoside (IPTG, 20 μg/ml, ForMedium), kanamycin (50 μg/ml, Sigma-Aldrich), or tetracycline (15–150 μg/ml, Sigma-Aldrich) when required. Strains were kept on plate at 4°C, or at −80°C in glycerol (25% v/v, Sigma-Aldrich).

### DNA methods and construction of plasmids

Genomic DNA was collected with the Puregene Yeast/Bact. Kit B (Qiagen). Synthetic genes and primers were obtained from IDT DNA. Genes encoding bacteriocins (ERS445055_00256 from *P. aeruginosa* NCTC10332, PFLQ8_1129 from *Pseudomonas fluorescens* Q8r1-96 (Landa et al., 2003) and PSPTO_0572 from *Pseudomonas syringae* pv. *tomato* DC3000) and putative immunity proteins, encoded downstream, were amplified by polymerase chain reaction (PCR) with Q5 polymerase (New England Biolabs), with a T100™ Thermal cycler (Bio-Rad). Q5 polymerase was used according to the supplier's specifications. All primers are listed in Table S2. PCR fragments were purified with the Genelute PCR clean-up kit (Sigma-Aldrich), and double digested with NcoI/XhoI (1 h at 37°C, New England Biolabs) in the case of the bacteriocin genes, and with PstI/XbaI (1 h at 37°C, New England Biolabs) in the case of the putative immunity genes. The immunity gene amplicon of *P. aeruginosa* NCTC10332 was digested with PstI/EcoRI (1 h at 37°C, New England Biolabs). Purified bacteriocin genes were ligated with T4 DNA ligase (1 h at 37°C, Invitrogen) in pET28a(+) and putative immunity genes in shuttle vector pJB3Tc20. Ligation products were transformed to *E. coli* DH5α via heat shock. Standard methods were used for the preparation of competent *E. coli* cells and heat shock transformation of *E. coli* (Green and Sambrook, 2012). Transformants were initially verified for the presence of insert by PCR with *Taq* polymerase (New England Biolabs), using conditions as specified by the supplier. Plasmids were subsequently harvested with the Genelute HP plasmid miniprep kit (Sigma-Aldrich) and sequence confirmed for the presence of correct insert (GATC Biotech, Constance, Germany). Cloned immunity genes encompassed a 19-bp upstream region containing the predicted ribosome binding sites. Resulting plasmids are summarized in Table S1. The pET28a constructs were transformed to *E. coli* BL21/DE3 via heat shock, and pJB3Tc20 constructs electroporated to PseuM-susceptible *Pseudomonas* strains, and selected on the proper medium. Immunity genes encoding a mutation in a conserved aspartate residue were constructed via splicing by overlap extension, using pCMPG6251 (encoding the putative immunity complement of PseuM_DC3000_), pCMPG6252 (complement of PseuM_NCTC10332_), and pCMPG6269 (complement of PseuM_Q8r1-96_) as a template.

### Expression and purification of recombinant bacteriocins

Recombinant carboxy-terminally His-tagged bacteriocins were generated in *E. coli* BL21(DE3) carrying pCMPG6248 (encoding PseuM from *P. syringae* DC3000), pCMPG6250 (encoding PseuM from *P. aeruginosa* NCTC10332) or pCMPG6271 (encoding PseuM from *P. fluorescens* Q8r1-96). Five milliliters of overnight cultures were transferred to 500 ml LB Erlenmeyer flasks and incubated at 37°C until OD_600_ reached 0.7. After, cultures were cooled and supplemented with IPTG (1 mM final concentration) and incubated at 20°C for 16 h. Next day, cells were harvested via centrifugation (20 min, 5000 g; Beckman X-15R) and frozen overnight (−20°C). Subsequently, the cell pellets were thawed, resuspended in lysis buffer (5 ml/g cell pellet; 300 mM NaCl, 50 mM NaH_2_PO_4_, 10 mM imidazole, pH 8.0) and sonicated (amplitude 20%, 10 cycles of 30 s on/off; Branson Digital Sonifier). Samples were treated with nuclease (0.01 U/μl, 37°C, 1 h; Invitrogen), remaining debris and insoluble proteins removed via centrifugation (30 min, 10000 g), and supernatants filtered (0.20 μm, Sarstedt). Soluble fractions containing the recombinant proteins were loaded on a 5-ml HisTrap HP column (GE Healthcare) and purified by nickel affinity chromatography with an Äkta Purifier (GE Healthcare). Matrix-bound proteins were eluted with a linear gradient of imidazole (10–500 mM) in lysis buffer. Fractions eluted at high imidazole concentrations were validated for the presence of recombinant protein, pooled and dialyzed to Tris buffer (50 mM, NaCl 200 mM, pH 7.5). Concentration of purified PseuMs was determined with a Genesys 10S UV-Vis Spectrophotometer (Thermo Scientific). Calculated extinction coefficients were 30035 mol/L^−1^ cm^−1^, 73340 mol/L^−1^ cm^−1^, and 32890 mol/L^−1^ cm^−1^ for the His-tagged PseuM bacteriocins from strains DC3000, NCTC10332, and Q8r1-96, respectively.

### Bacteriocin assay

Bacteriocin activity was determined via spot assay. Filter-sterilized 20-μl volumes of purified recombinant proteins (1 mg/ml) were spotted onto bacterial cell lawns. After, samples were air-dried and incubated overnight. Dialysis buffer was used as a negative control. Following day, petri dishes were scored for zones lacking bacterial growth (halos).

### Nucleotide accession number

GenBank accession number of the nucleotide sequence of the *pseuM-pmiA* pair from *P. putida* RW10S2 (Rokni-Zadeh et al., 2012) is KX086738.

## Results

### *In silico* search for candidate genes providing immunity to *Pseudomonas* colicin M-like bacteriocins

Based on the genetic architecture of modular bacteriocins in Gram-negatives, it is anticipated that bacteriocin immunity genes are likely to be found in proximity of their corresponding toxin genes. By scrutiny of their genomic contexts we observed that the characterized *Pseudomonas* colicin M-like bacteriocin genes, as well as several other predicted PseuM-encoding genes (Figure S1A), are consistently followed on the same DNA strand—at variable distances but within <200 nucleotides—by an open reading frame, but infrequently annotated as a coding region (Table S3). Typically, G+C content of these regions (~1.8 kb) is significantly lower (41–52%) than the *Pseudomonas* genome averages (*P. aeruginosa*: 66.2%, 1647 genomes; *P. fluorescens*: 60.2%, 92 genomes; *P. syringae*: 58.7%, 153 genomes). None of the deduced protein sequences contains the YebF domain signature (Pfam PF13995) as found in Cmi and the putative pectocin M immunity proteins.

In 14 *Pseudomonas* strains, mostly *P. syringae* pathovars, the encoded proteins (encompassing eight unique but very similar sequences of ~100 AAs) show only borderline homology with the burkhocin-associated BmiB immunity proteins (12–24% pairwise AA identity). However, these pseudomonad proteins, designated PmiBs, share with BmiBs two perfectly conserved cysteines (Figure S2). The PmiB and BmiB proteins are predicted to be translocated to the periplasm, albeit this appears to be achieved in different ways: instead of the BmiB amino-terminal membrane-anchoring segment or Sec-dependent cleavable signal sequence, a cleavable lipoprotein signal sequence typifies PmiB. This common feature is reflected in the phylogeny of ColM domains that reveals a well-separated clade harboring both the burkhocins and this small subset of PseuM proteins (Figure S1B).

**Figure 1:**
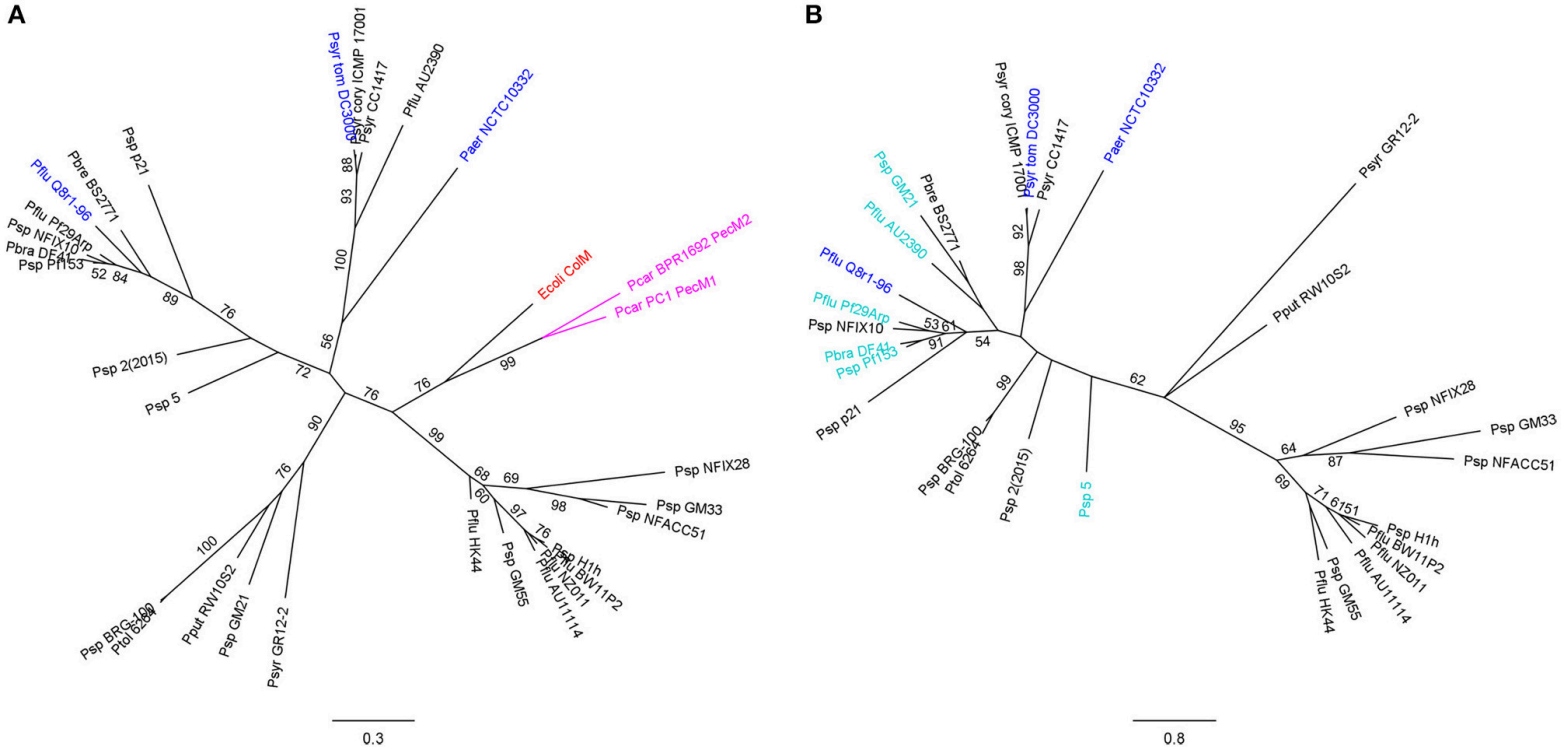
**Maximum likelihood phylogenetic trees of ColM domains from *pmiA*-associated PseuMs and from related γ-proteobacterial bacteriocins (A)**, and of associated *Pseudomonas* PmiA immunity proteins **(B)**. Characterized PseuM bacteriocins and cognate PmiAs are colored blue, colicin M (ColM) from *E. coli* is shown in red, and colicin M domain-containing pectocins (PecM1 and PecM2) in pink. PmiAs that were studied for promiscuity are shown in teal. The scale bars represent 0.3 **(A)** and 0.8 **(B)** substitutions per site. Bootstrap values (percentages of 1000 replicates) higher than 50 are shown at the branches. **(A)** For highly homologous colicin M domains (>95% pairwise AA identity), only one representative was included for phylogenetic tree construction. Species abbreviations: Ecoli, *Escherichia coli*; Paer, *Pseudomonas aeruginosa*; Pbra, *Pseudomonas brassicacearum*; Pbre, *Pseudomonas brenneri*; Pcar, *Pectobacterium carotovorum*; Pflu, *Pseudomonas fluorescens*; Pput, *Pseudomonas putida*; Psp, *Pseudomonas* sp.; Psyr, *Pseudomonas syringae*; Psyr cory, *P. syringae* pv. *coryli*; Psyr tom, *P. syringae* pv. *tomato*; Ptol, *Pseudomonas tolaasii*.

**Figure 2:**
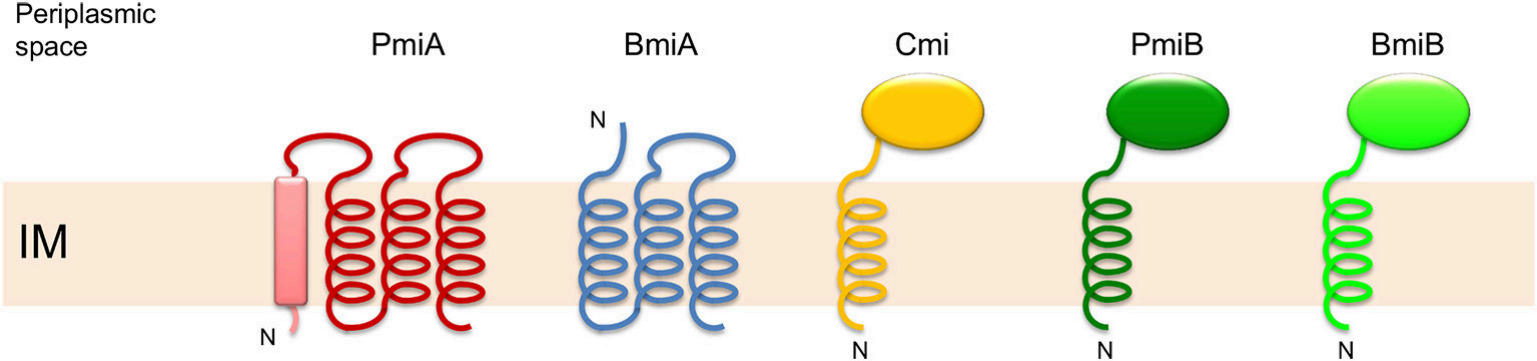
**Membrane topology model of integral membrane proteins PmiA and BmiA, and membrane-anchored proteins Cmi, PmiB, and BmiB, providing immunity to the activity of the ColM domain**. PmiA from *Pseudomonas* has four predicted transmembrane helices, the first of which may represent a Sec- or Tat-dependent signal sequence (box). BmiA from *Burkholderia* carries three transmembrane helices. Cmi has a periplasmic YebF domain and is anchored in the inner membrane (IM) via an amino-terminal helix. PmiB and BmiB are equally anchored in the IM, and carry homologous periplasmic domains. “N” depicts the amino-terminus.

The majority of PseuM-derived ColM domains constitutes a separate clade of much more diversified sequences that are evolutionarily related to the corresponding domains in colicin M and colicin M domain-containing pectocins (Figure 1A, Figure S1B). In this second subset of PseuM-bacteriocinogenic strains the bacteriocin gene is linked with a gene encoding a protein unrelated to PmiB (~137 AAs; Figure S3). This protein family, designated PmiA, features four transmembrane helices, of which the first is predicted to represent a candidate Sec- or Tat-dependent cleavable signal sequence (Figure 2). The inferred topology, with two moieties facing the periplasm and two stretches (including the carboxy-terminus) residing in the cytoplasm, is reminiscent of the one adopted by BmiA proteins, albeit the latter lack an equivalent secretory signal sequence (Ghequire and De Mot, 2015b). No sequence homology can be detected between BmiAs and PmiAs however. Notably, four phytopathogenic strains of the *P. syringae* group each carry two unlinked PseuM-encoding paralogues, one from each PseuM clade and clustered with, respectively, a *pmiA* and *pmiB* gene. The low level of sequence identity between these bacteriocins (~24%) combined with the different nature of the respective candidate immunity proteins argues against a common ancestry and favors their independent acquisition.

In different strains, the *pseuM-pmiA* gene pair has been recruited to diverse genomic locations. Several of these regions correspond to mobile elements. In *P. aeruginosa*, the gene couple generally occurs on ExoU island A, as has previously been noted for the *pseuM* of *P. aeruginosa* JJ692 (Barreteau et al., 2009). In some strains from the *P. syringae* group, such as isolates 642 and LMG 2367, *pseuM-pmiA* combinations appear as a cargo gene couple in a *trpE*/*trpG*-integrated Rp4 tailocin cluster (Hockett et al., 2015). In other *P. syringae* pathovar strains, such as DC3000 and ICMP 3923, the cargo is equally part of a Rp4-type sequence, although incomplete and apparently linked with diverged sequences. In some *P. fluorescens* group strains, such as *Pseudomonas brassicacearum* NFM421 and 51MFVCI2.1, and *P. fluorescens* Q8r1-96, the gene pair has been recruited by a Rp4 tailocin cluster as well, sandwiched between *mutS* and *cinA* (Ghequire et al., 2015; Hockett et al., 2015). For other strains, such as *P. veronii* R4, *Pseudomonas* sp. BRG-100 and *Pseudomonas tolaasii* 6264, regions flanking the *pseuM-pmiA* couples correspond to an unassigned prophage. The presence of bacteriocin cargo genes on mobile elements has previously been noted for lectin-like bacteriocins embedded in *Pseudomonas* Rp2 tailocin clusters (Ghequire et al., 2015).

**Figure 3:**
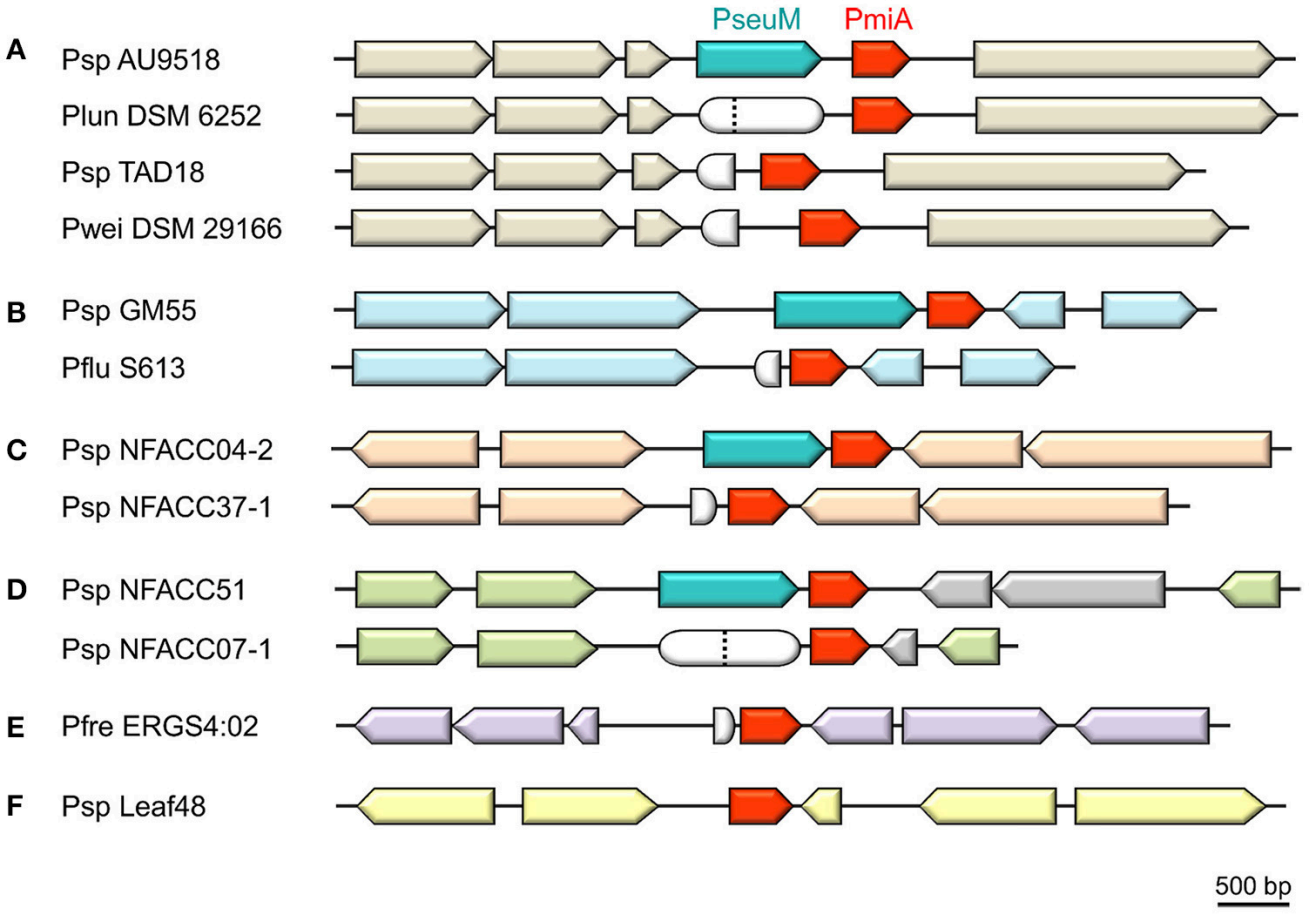
**Genomic context of orphan *pmiA* genes in pseudomonads**. Syntenic regions carrying a *pmiA* homolog associated with a full-length PseuM-encoding gene are shown for *Pseudomonas* sp. strains AU9518 **(A)**, GM55 **(B)**, NFACC04-2 **(C)**, and NFACC51 **(D)**. Synteny between these predicted bacteriocin producers and phylogenetically related cheaters lacking a (functional) bacteriocin gene is highlighted by upstream and downstream ORFs (arrows) shown in the same color. The gray-colored ORFs **(D)** reflect a local lack of synteny. Remnants of the respective *pseuM* genes are represented by white-colored rounded shapes corresponding to a frameshifted *pseuM* (position marked by a dotted vertical line) or a truncated residual N- or C-terminal fragment. For *Pseudomonas* strains ERGS4:02 **(E)** and Leaf48 **(F)**, no strain with a closely related *pmiA* homolog and syntenic flanking region could be identified. Species abbreviations: Pfre, *Pseudomonas frederikbergensis*; Plun, *Pseudomonas lundensis*; Pwei, *Pseudomonas weihenstephanensis*. Other abbreviations are as in Figure 1.

Some *Pseudomonas* strains carry a *pmiA* gene that is not linked to a cognate *pseuM* gene. The presence of *pseuM* remnant sequences upstream of such a *pmiA* orphan indicates that these strains may represent so-called cheaters that have lost the capacity of bacteriocin production but retain the associated immune function (Figure 3). For instance, in the type strain *Pseudomonas lundensis* (DSM 6252; De Jonghe et al., 2011) a frameshift impairs functionality of a *pseuM* gene that has remained intact in *Pseudomonas* sp. AU9518. From the equivalent genomic region of type strain *Pseudomonas weihenstephanensis* (DSM 291166; von Neubeck et al., 2016) and some related strains (TAD18; TAA207 and DSM 28140, not included in Figure 3), most of the 3′ region of a *pseuM* gene has been deleted, while their *pmiA* genes are well-conserved (>92% AA identity). No obvious *pseuM* sequences remain adjacent to the *pmiA* gene of Arabidopsis isolate *Pseudomonas* sp. Leaf48 (Bai et al., 2015), that is flanked by prophage genes.

### PmiA proteins are cognate PseuM immunity partners

Previously described PseuM bacteriocins from *P. aeruginosa* NCTC10332 (PaeM), *P. fluorescens* Q8r1-96 (PflM), and *P. syringae* pv. *tomato* DC3000 (PsyM), each with a downstream *pmiA* gene (as identified by *in silico* analysis described in Section *In silico* Search for Candidate Genes Providing Immunity to *Pseudomonas* Colicin M-like Bacteriocins), were recombinantly expressed in *E. coli* and the His-tagged proteins purified by Ni-NTA affinity chromatography. Strain NCTC10332 encodes a PseuM bacteriocin that shares 90% AA identity with PaeM from *P. aeruginosa* JJ692 (Barreteau et al., 2009). Antibacterial activity of the recombinant proteins was challenged via spot-on-lawn assay against a panel of *Pseudomonas* strains, and strains with a clear halo susceptibility phenotype were selected for further experiments. Such indicator strains identified for PaeM, PflM, and PsyM were *P. aeruginosa* CPHL12447, *P. fluorescens* F113, and *P. syringae* pv. *lachrymans* LMG 5456 (Grinter et al., 2012b), respectively.

Next, the putative immunity genes from strains NCTC10332, Q8r1-96 and DC3000 were cloned in shuttle vector pJB3Tc20, and introduced in the corresponding PseuM-susceptible strains. Transformants were tested for altered PseuM sensitivity, using strains carrying empty vectors as negative controls (Table 1). When equipped with the cognate *pmiA*, transformants became fully insensitive to the respective bacteriocin (tested at 1 mg/ml by spot assay). When indicator strains were provided with either of the other two immunity genes, no diminished PseuM inhibition was observed. Together this indicates that PmiAs are cognate immunity partners of the bacteriocins they are associated with. Purified membrane fragments with the gene product of *pmiA*_*DC*3000_ were analyzed via Maldi-MSMS (Poetsch et al., 2008), but no peptide fragments assignable to the PmiA of interest could be retrieved (data not shown).

**Table 1:** **Heterologous expression of *pmiA* genes in PseuM-susceptible strains**.

**Indicator strain**	**PmiA immunity protein**		**PseuM bacteriocin**		
	**Species**	**Gene product**	**PaeM *P. aeruginosa* NCTC10332**	**PflM *P. fluorescens* Q8r1-96**	**PsyM *P. syringae* pv. tomato DC3000**
*P. aeruginosa* CPHL12447	*P. aeruginosa*	PmiA_NCTC10332_	−		
		PmiA^*^_NCTC10332_	−		
	*P. fluorescens*	PmiA_Q8r1-96_	+		
	*P. syringae* pv. *tomato*	PmiA_DC3000_	+		
	NA	Control	+		
*P. fluorescens* F113	*P. aeruginosa*	PmiA_NCTC10332_		+	
	*P. brassicacearum*	PmiA_DF41_		−	
	*P. fluorescens*	PmiA_Q8r1-96_		−	
		PmiA^*^_Q8r1-96_		−	
		PmiA_AU2390_		+	
		PmiA_Pf29Arp_		−	
	*P. syringae* pv. *syringae*	PmiA_DC3000_		+	
	*Pseudomonas* sp.	PmiA_5_		T	
		PmiA_GM21_		+	
		PmiA_Pf153_		−	
	NA	Control		+	
*P. syringae* LMG 5456	*P. aeruginosa*	PmiA_NCTC10332_			+
	*P. syringae* pv. *tomato*	PmiA_DC3000_			−
		PmiA^*^_DC3000_			−
	NA	Control			+

*Indicator strains were transformed with shuttle vector pJB3Tc20 (control, without insert) or with pJB3Tc20-derived plasmids equipped with different pmiA genes, and tested for (altered) susceptibility to their respective PseuM via bacteriocin spot assay. +, sensitive; −, insensitive; T, turbid halo. The species used as source of immunity protein are specified with the respective strain names indicated as PmiA subscript (NA, not applicable). Asterisks define mutated immunity proteins (Asp to Ala). Strain LMG 5456 carrying a plasmid equipped with pmiA_Q8r1−96_ could not be obtained*.

**Figure 4:**
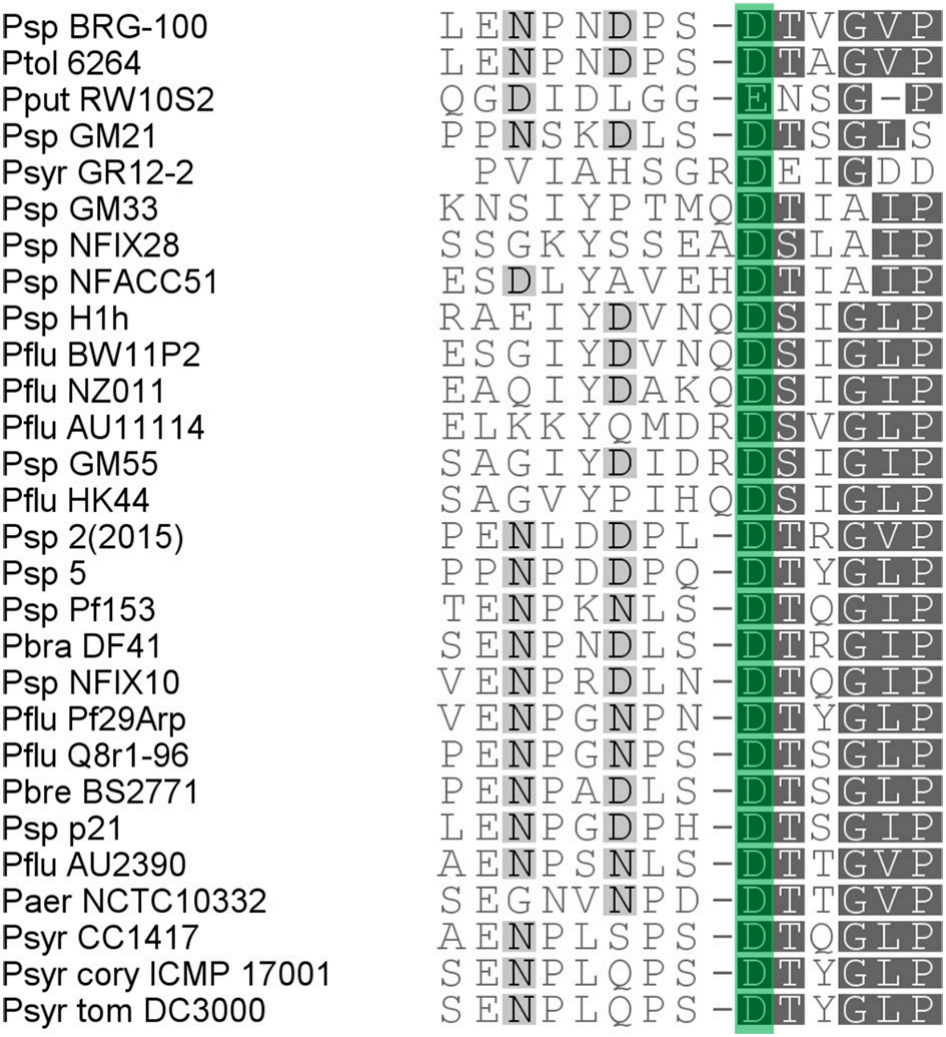
**Multiple sequence alignment of the semi-conserved stretch in PmiA proteins, predicted to be oriented toward the periplasmic space**. *Pseudomonas* species abbreviations are as in Figure 1. Gray shading reflects the degree of conservation. The conserved Asp residue that was mutated is boxed in green.

Since colicin M and colicin M-like bacteriocins domains exert their action in the periplasm, it is plausible that (at least part of) the periplasm-exposed moieties of PmiA proteins participate in the immunity function, directly or indirectly impeding the catalytic function of the ColM domain. Conserved residues in the immunity module may be critical in providing such property. In the mature PmiA proteins, a short well-conserved stretch with consensus motif D[T/S]XGXP precedes the second predicted transmembrane segment (Figure 4, Figure S3). No obvious sequence conservation among PmiA proteins can be detected for the shorter second periplasm-exposed loop. To further scrutinize the possible role of the first periplasmic stretch, the conserved Asp residue was mutated to Ala in the PmiAs of strains NCTC10332, Q8r1-96 and DC3000. Upon introduction of the PmiA variants in the respective indicator strains, the immunity phenotype to the respective PseuMs was evaluated. Halos were absent for all three indicator strains after bacteriocin spotting, showing the same phenotype as caused by the native immunity gene products (Table 1). This suggests that the conserved Asp residue is not instrumental to PmiA's immunity function.

### Immune promiscuity among sequence-diverged PmiAs

Noteworthy, sequence divergence of the PseuM-PmiA toxin-immunity pairs seems to be most pronounced among strains belonging to the *P. fluorescens* group. In contrast, for *P. syringae* pathovar isolates, high conservation of the ColM domain (at least 94% pairwise AA identity; Figures S1A,B) is reflected in well-conserved immunity proteins (at least 84% pairwise AA identity). Previously, it was demonstrated that immunity proteins sharing high homology may provide pyocin-silencing functionality of another partner (Dingemans et al., 2016).

To examine to what extent sequence divergence limits the immune function, PmiA homologs from the cluster with *P. fluorescens* Q8r1-96 were selected (*P. brassicacearum* DF41, *P. fluorescens* Pf29Arp, and *Pseudomonas* sp. Pf153), as well as some more distant PmiAs (*Pseudomonas* sp. strains 5 and GM21, *P. fluorescens* AU2390; Figure 1B). The PmiAs from the Q8r1-96 cluster share 36–40% AA identity with PmiA_Q8r1-96_. This level of sequence conservation stems mainly from the periplasm-exposed regions as well as the transmembrane helices, whereas the amino-terminal signal sequence/transmembrane helix and the cytoplasm-exposed stretches exhibit extensive sequence divergence. By comparison, the ColM domains of the PseuMs of strains DF41, Pf29Arp and Pf153, display considerably higher sequence conservation (~69% AA identity) with PflM. Pairwise identity with the more distant PmiAs ranges from 18 to 31%, corresponding with more diversified ColM domains (35–46% AA identity with PflM).

These six *pmiA* genes were cloned in pJB3Tc20 and introduced in *P. fluorescens* F113, and tested for their capacity to protect from PflM activity, using empty vector and the native *pmiA*_Q8r196_ immunity gene as controls for susceptibility and immunity, respectively (Table 1). Despite only moderate pairwise identities to PmiA_Q8r1-96_, PmiA_DF41_, PmiA_Pf153_, and PmiA_Pf29Arp_ provided full cross-immunity to PflM. The more distantly related PmiAs from strains AU2390 and GM21 (with 30.9 and 23.4% AA identity, respectively) could not immunize cells against the bacteriocin, resulting in a similar phenotype as the PmiAs from *P. aeruginosa* NCTC10332 and *P. syringae* DC3000, and the control. Despite barely showing homology with PmiA_Q8r1-96_ (18.4% AA identity), PmiA from *Pseudomonas* sp. 5 conferred an intermediate immunity phenotype (turbid halo).

## Discussion

**Figure 5:**
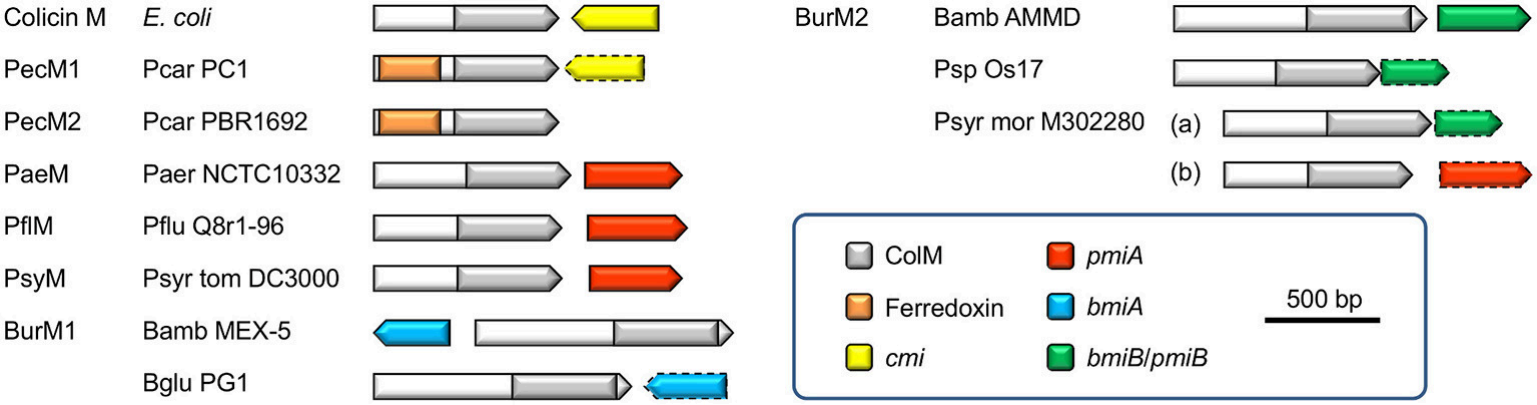
**Schematic gene organizations of ColM domain-containing bacteriocins and (putative) immunity genes (if present)**. The arrows correspond with the gene orientations and the color legend describes the indicated domains and type of immunity gene. Predicted immunity genes, based on homology with characterized immunity factors, are delineated by a dashed line. Species abbreviations are as in Figure 1. Bamb, *Burkholderia ambifaria*; Bglu, *Burkholderia glumae*.

In this study we demonstrated that cognate immunity to a large subset of ColM domain-containing *Pseudomonas* bacteriocins is provoked by the poorly conserved transmembrane protein PmiA, unrelated in sequence and topology to the Cmi protein providing immunity to colicin M in *E. coli*. The *pmiA* gene, consistently located downstream of *pseuM* on the same strand, is rarely annotated (Table S3). Previously, immunity to ColM domains in *Burkholderia* burkhocins was equally associated with a transmembrane protein (BmiA; Ghequire and De Mot, 2015b). However, since ColM domains from *bmiA*- and *pmiA*-associated bacteriocins constitute phylogenetically distant clades, the immunity mechanism from these integral membrane proteins may be different. Further support for the existence of such evolutionarily distinct lipid II-targeting bacteriocin subsets is provided by the observation that a minor group of pseudomonad genes shares a similar immunity gene, as well as a similar ColM-domain-encoding gene, with *Burkholderia*. The gene organization of different ColM domain-hosting bacteriocins and their (putative) immunity genes is summarized in Figure 5.

A conserved Asp residue located in a semi-conserved periplasm-exposed stretch proved not pivotal for the immunity phenotype of PmiA. Possibly, it is required for structural integrity or stability of the immunity protein, and structural analysis can give more insight in this issue. Since the periplasm-exposed moieties of PmiA likely contribute to immunity, it was investigated whether cross-immunity between proteins with different extent of sequence conservation in those regions, may occur. Since this was found to be the case, even between PmiAs sharing <40% pairwise amino acid identity, it will be of future interest to determine which residues or structural elements actually contribute to the immunity phenotype. This will render a first indication on how and where the immunity mechanism to this toxin module may be provoked. Previously, attempts to co-crystallize colicin M and Cmi to enlighten how Cmi neutralizes ColM-mediated toxicity proved unsuccessful (Usón et al., 2012).

From an evolutionary point of view, promiscuous protection by immunity genes sharing low homology may have far-reaching consequences toward competitive advantage for producer strains. It may explain why genome analysis in pseudomonads indicated that PseuMs are rather rare, as compared to DNase and RNase bacteriocins (Ghequire and De Mot, 2014). In contrast, immunity to the latter bacteriocins is provided by immunity proteins forming very specifically high-affinity bacteriocin-immunity complexes, stabilized by conserved hydrogen bonds and hydrophobic interactions (Li et al., 2004; Levin et al., 2009; Meenan et al., 2010; Joshi et al., 2015; Kleanthous et al., 2016). PseuM bacteriocin production may therefore provide limited benefit in most environments, and hence result in lower selection pressure to maintain these bacteriocins in genomes. In addition to this, genome mining also indicated that orphan *pmiA*-like genes occasionally occur in *Pseudomonas* genomes, although it remains to be investigated whether these enable cheating. A similar observation was made for *bmiA* genes in *Burkholderia* (Ghequire and De Mot, 2015b) and S-type pyocin immunity genes (Ghoul et al., 2015).

## Author contributions

MG, RD conceived and designed the experiments; MG and LK performed the experiments; MG, LK, and RD analyzed the data; MG and RD wrote the paper; all authors approved the final version of the manuscript.

## Funding

MG is the recipient of a postdoctoral fellowship from FWO-Vlaanderen (12M4615N).

### Conflict of interest statement

The authors declare that the research was conducted in the absence of any commercial or financial relationships that could be construed as a potential conflict of interest.

## References

[B1] Bai Y. Müller D. B. Srinivas G. Garrido-Oter R. Potthoff E. Rott M.. (2015). Functional overlap of the *Arabidopsis* leaf and root microbiota. Nature 528, 364–369. 10.1038/nature16192 26633631

[B2] Barreteau H. Bouhss A. Fourgeaud M. Mainardi J. L. Touzé T. Gérard F.. (2009). Human- and plant-pathogenic *Pseudomonas* species produce bacteriocins exhibiting colicin M-like hydrolase activity towards peptidoglycan precursors. J. Bacteriol. 191, 3657–3664. 10.1128/JB.01824-08 19346308 PMC2681890

[B3] Barreteau H. Tiouajni M. Graille M. Josseaume N. Bouhss A. Patin D.. (2012). Functional and structural characterization of PaeM, a colicin M-like bacteriocin produced by *Pseudomonas aeruginosa*. J. Biol. Chem. 287, 37395–37405. 10.1074/jbc.M112.406439 22977250 PMC3481336

[B4] Cascales E. Buchanan S. K. Duché D. Kleanthous C. Lloubès R. Postle K.. (2007). Colicin biology. Microbiol. Mol. Biol. Rev. 71, 158–229. 10.1128/MMBR.00036-06 17347522 PMC1847374

[B5] De Jonghe V. Coorevits A. Van Hoorde K. Messens W. Van Landschoot A. De Vos P.. (2011). Influence of storage conditions on the growth of *Pseudomonas* species in refrigerated raw milk. Appl. Environ. Microbiol. 77, 460–470. 10.1128/AEM.00521-10 21115713 PMC3020527

[B6] Dingemans J. Ghequire M. G. Craggs M. De Mot R. Cornelis P. (2016). Identification and functional analysis of a bacteriocin, pyocin S6, with ribonuclease activity from a *Pseudomonas aeruginosa* cystic fibrosis clinical isolate. Microbiologyopen 5, 413–423. 10.1002/mbo3.339 26860427 PMC4905994

[B7] Gérard F. Brooks M. A. Barreteau H. Touzé T. Graille M. Bouhss A.. (2011). X-ray structure and site-directed mutagenesis analysis of the *Escherichia coli* colicin M immunity protein. J. Bacteriol. 193, 205–214. 10.1128/JB.01119-10 21037007 PMC3019942

[B8] Gerstmans H. Rodríguez-Rubio L. Lavigne R. Briers Y. (2016). From endolysins to Artilysin®s: novel enzyme-based approaches to kill drug-resistant bacteria. Biochem. Soc. Trans. 44, 123–128. 10.1042/BST20150192 26862197

[B9] Ghequire M. G. De Mot R. (2014). Ribosomally encoded antibacterial proteins and peptides from *Pseudomonas*. FEMS Microbiol. Rev. 38, 523–568. 10.1111/1574-6976.12079 24923764

[B10] Ghequire M. G. De Mot R. (2015a). The tailocin tale: peeling off phage tails. Trends Microbiol. 23, 587–590. 10.1016/j.tim.2015.07.011 26433692

[B11] Ghequire M. G. De Mot R. (2015b). Distinct colicin M-like bacteriocin-immunity pairs in *Burkholderia*. Sci. Rep. 5:17368. 10.1038/srep17368 26610609 PMC4661593

[B12] Ghequire M. G. Dillen Y. Lambrichts I. Proost P. Wattiez R. De Mot R. (2015). Different ancestries of R tailocins in rhizospheric *Pseudomonas* isolates. Genome Biol. Evol. 7, 2810–2828. 10.1093/gbe/evv184 26412856 PMC4684702

[B13] Ghequire M. G. Dingemans J. Pirnay J. P. De Vos D. Cornelis P. De Mot R. (2014). O serotype-independent susceptibility of *Pseudomonas aeruginosa* to lectin-like pyocins. Microbiologyopen 3, 875–884. 10.1002/mbo3.210 25224846 PMC4263511

[B14] Ghequire M. G. Garcia-Pino A. Lebbe E. K. Spaepen S. Loris R. De Mot R. (2013). Structural determinants for activity and specificity of the bacterial toxin LlpA. PLoS Pathog. 9:e1003199. 10.1371/journal.ppat.1003199 23468636 PMC3585409

[B15] Ghoul M. West S. A. Johansen H. K. Molin S. Harrison O. B. Maiden M. C.. (2015). Bacteriocin-mediated competition in cystic fibrosis lung infections. Proc. Biol. Sci. 282, 1814. 10.1098/rspb.2015.0972 26311664 PMC4571691

[B16] Green M. R. Sambrook J. (2012). Molecular Cloning: A Laboratory Manual. New York, NY: Cold Spring Harbor.

[B17] Grinter R. Josts I. Zeth K. Roszak A. W. McCaughey L. C. Cogdell R. J.. (2014). Structure of the atypical bacteriocin pectocin M2 implies a novel mechanism of protein uptake. Mol. Microbiol. 93, 234–246. 10.1111/mmi.12655 24865810 PMC4671253

[B18] Grinter R. Milner J. Walker D. (2012a). Ferredoxin containing bacteriocins suggest a novel mechanism of iron uptake in *Pectobacterium* spp. PLoS ONE 7:e33033. 10.1371/journal.pone.0033033 22427936 PMC3302902

[B19] Grinter R. Roszak A. W. Cogdell R. J. Milner J. J. Walker D. (2012b). The crystal structure of the lipid II-degrading bacteriocin syringacin M suggests unexpected evolutionary relationships between colicin M-like bacteriocins. J. Biol. Chem. 287, 38876–38888. 10.1074/jbc.M112.400150 22995910 PMC3493929

[B20] Gross P. Braun V. (1996). Colicin M is inactivated during import by its immunity protein. Mol. Gen. Genet. 251, 388–396. 10.1007/BF02172531 8676883

[B21] Hockett K. L. Renner T. Baltrus D. A. (2015). Independent co-option of a tailed bacteriophage into a killing complex in *Pseudomonas*. MBio 6:e00452. 10.1128/mBio.00452-15 26265717 PMC4542187

[B22] Holt K. E. Thieu Nga T. V. Thanh D. P. Vinh H. Kim D. W. Vu Tra M. P.. (2013). Tracking the establishment of local endemic populations of an emergent enteric pathogen. Proc. Natl. Acad. Sci. U.S.A. 110, 17522–17527. 10.1073/pnas.1308632110 24082120 PMC3808646

[B23] Jones D. T. Taylor W. R. Thornton J. M. (1992). The rapid generation of mutation data matrices from protein sequences. Comput. Appl. Biosci. 8, 275–282. 10.1093/bioinformatics/8.3.275 1633570

[B24] Joshi A. Grinter R. Josts I. Chen S. Wojdyla J. A. Lowe E. D.. (2015). Structures of the ultra-high-affinity protein-protein complexes of pyocins S2 and AP41 and their cognate immunity proteins from *Pseudomonas aeruginosa*. J. Mol. Biol. 427, 2852–2866. 10.1016/j.jmb.2015.07.014 26215615 PMC4548480

[B25] Kleanthous C. Klein A. Wojdyla J. Joshi A. Josts I. McCaughey L. C. (2016). Structural and biophysical analysis of nuclease proteins antibiotics. Biochem. J. 473, 2799–2812. 10.1042/BCJ20160544 27402794 PMC5264503

[B26] Landa B. B. Mavrodi D. M. Thomashow L. S. Weller D. M. (2003). Interactions between strains of 2,4-diacetylphloroglucinol-producing *Pseudomonas fluorescens* in the rhizosphere of wheat. Phytopathology 93, 982–994. 10.1094/PHYTO.2003.93.8.982 18943865

[B27] Levin K. B. Dym O. Albeck S. Magdassi S. Keeble A. H. Kleanthous C.. (2009). Following evolutionary paths to protein-protein interactions with high affinity and selectivity. Nat. Struct. Mol. Biol. 16, 1049–1055. 10.1038/nsmb.1670 19749752

[B28] Li W. Keeble A. H. Giffard C. James R. Moore G. R. Kleanthous C. (2004). Highly discriminating protein-protein interaction specificities in the context of a conserved binding energy hotspot. J. Mol. Biol. 337, 743–759. 10.1016/j.jmb.2004.02.005 15019791

[B29] McCaughey L. C. Grinter R. Josts I. Roszak A. W. Waløen K. I. Cogdell R. J.. (2014). Lectin-like bacteriocins from *Pseudomonas* spp. utilise D-rhamnose containing lipopolysaccharide as a cellular receptor. PLoS Pathog. 10:e1003898. 10.1371/journal.ppat.1003898 24516380 PMC3916391

[B30] McCaughey L. C. Josts I. Grinter R. White P. Byron O. Tucker N. P.. (2016). Discovery, characterisation and *in vivo* activity of pyocin SD2, a protein antibiotic from *Pseudomonas aeruginosa*. Biochem. J. 473, 2345–2358. 10.1042/BCJ20160470 27252387 PMC4964976

[B31] Meenan N. A. Sharma A. Fleishman S. J. Macdonald C. J. Morel B. Boetzel R.. (2010). The structural and energetic basis for high selectivity in a high-affinity protein-protein interaction. Proc. Natl. Acad. Sci. U.S.A. 107, 10080–10085. 10.1073/pnas.0910756107 20479265 PMC2890441

[B32] Olschläger T. Braun V. (1987). Sequence, expression, and localization of the immunity protein for colicin M. J. Bacteriol. 169, 4765–4769. 10.1128/jb.169.10.4765-4769.1987 2820942 PMC213852

[B33] Olschläger T. Schramm E. Braun V. (1984). Cloning and expression of the activity and immunity genes of colicins B and M on ColBM plasmids. Mol. Gen. Genet. 196, 482–487. 10.1007/BF00436196 6094976

[B34] Olschläger T. Turba A. Braun V. (1991). Binding of the immunity protein inactivates colicin M. Mol. Microbiol. 5, 1105–1111. 10.1111/j.1365-2958.1991.tb01883.x 1956288

[B35] Poetsch A. Schlüsener D. Florizone C. Eltis L. Menzel C. Rögner M.. (2008). Improved identification of membrane proteins by MALDI-TOF MS/MS using vacuum sublimated matrix spots on an ultraphobic chip surface. J. Biomol. Tech. 19, 129–138. 19137096 PMC2361163

[B36] Rokni-Zadeh H. Li W. Sanchez-Rodriguez A. Sinnaeve D. Rozenski J. Martins J. C.. (2012). Genetic and functional characterization of cyclic lipopeptide white-line-inducing principle (WLIP) production by rice rhizosphere isolate *Pseudomonas putida* RW10S2. Appl. Environ. Microbiol. 78, 4826–4834. 10.1128/AEM.00335-12 22544260 PMC3416372

[B37] Schulz S. Stephan A. Hahn S. Bortesi L. Jarczowski F. Bettmann U.. (2015). Broad and efficient control of major foodborne pathogenic strains of *Escherichia coli* by mixtures of plant-produced colicins. Proc. Natl. Acad. Sci. U.S.A. 112, E5454–E5460. 10.1073/pnas.1513311112 26351689 PMC4603501

[B38] Usón I. Patzer S. I. Rodríguez D. D. Braun V. Zeth K. (2012). The crystal structure of the dimeric colicin M immunity protein displays a 3D domain swap. J. Struct. Biol. 178, 45–53. 10.1016/j.jsb.2012.02.004 22366279

[B39] von Neubeck M. Huptas C. Glück C. Krewinkel M. Stoeckel M. Stressler T.. (2016). *Pseudomonas helleri* sp. nov. and *Pseudomonas weihenstephanensis* sp. nov., isolated from raw cow's milk. Int. J. Syst. Evol. Microbiol. 66, 1163–1173. 10.1099/ijsem.0.000852 26675012

