# Supplementary material for: Novel Immunity Proteins Associated with Colicin M-like Bacteriocins Exhibit Promiscuous Protection in Pseudomonas
Source: Front Microbiol. 2017 Jan 30;8:93. doi: 10.3389/fmicb.2017.00093 (PMC5277000; doi:10.3389/fmicb.2017.00093)
Supplement: Supplementary file 1 [file DataSheet1.pdf]

Supplementary Material

Novel immunity proteins associated with colicin M-like bacteriocins exhibit promiscuous protection in *Pseudomonas*

Maarten G. K. Ghequire[1,2]*, Lieselore Kemland[1], René De Mot[1]

[1]Centre of Microbial and Plant Genetics, KU Leuven, 3001 Heverlee, Belgium
[2]National Institute of Diabetes and Digestive and Kidney Diseases, National Institutes of Health, Bethesda, MD, USA

*** Correspondence:** Maarten Ghequire: maarten.ghequire@biw.kuleuven.be

Supplementary Table S1. Strains and plasmids used in this study.

Plasmid/strain	Purpose of use	Reference
pET28a	pBR322 origin, His-tag/thrombin/T7 tag ; Km^R	Novagen
pJB3Tc20	Broad-host-range cloning vector; Ap^R, Tc^R	(Blatny *et al.*, 1997)
pCMPG6248	pET28a with 827-bp PCR-amplified fragment containing *pseuM* from *P. syringae* DC3000, cloned in NcoI/XhoI	This study
pCMPG6250	pET28a with 866-bp PCR-amplified fragment containing *pseuM* from *P. aeruginosa* NCTC10332, cloned in NcoI/XhoI	This study
pCMPG6251	pJB3Tc20 with 445-bp PCR-amplified fragment containing *pmiA* from *P. syringae* DC3000, cloned in PstI/XbaI	This study
pCMPG6252	pJB3Tc20 with 467-bp PCR-amplified fragment containing *pmiA* from *P. aeruginosa* NCTC10332, cloned in PstI/EcoRI	This study
pCMPG6266	Mutant construct of pCMPG6251; D36A variant of the encoded PmiA	This study
pCMPG6267	Mutant construct of pCMPG6252; D34A variant of the encoded PmiA	This study
pCMPG6269	pJB3Tc20 with 504-bp PCR-amplified fragment containing *pmiA* from *P. fluorescens* Q8r1-96, cloned in PstI/XbaI	This study
pCMPG6270	Mutant construct of pCMPG6269; D39A variant of the encoded PmiA	This study
pCMPG6271	pET28a with 812-bp PCR-amplified fragment containing *pseuM* from *P. fluorescens* Q8r1-96, cloned in NcoI/XhoI	This study
pCMPG6272	pJB3Tc20 with 478-bp PCR-amplified fragment containing *pmiA* from *Pseudomonas* sp. 5, cloned in PstI/XbaI	This study
pCMPG6273	pJB3Tc20 with 465-bp PCR-amplified fragment containing *pmiA* from *Pseudomonas* sp. Pf153, cloned in PstI/XbaI	This study
pCMPG6274	pJB3Tc20 with 459-bp PCR-amplified fragment containing *pmiA* from *P. fluorescens* Pf29Arp, cloned in PstI/XbaI	This study
pCMPG6275	pJB3Tc20 with 497-bp PCR-amplified fragment containing *pmiA* from *P. brassicacearum* DF41, cloned in PstI/XbaI	This study
pCMPG6276	pJB3Tc20 with 466-bp PCR-amplified fragment containing *pmiA* from *Pseudomonas* sp. GM21, cloned in PstI/XbaI	This study
pCMPG6277	pJB3Tc20 with 456-bp PCR-amplified fragment containing *pmiA* from *P. fluorescens* AU2390, cloned in PstI/XbaI	This study
E. coli BL21(DE3)	Expression host	VWR
E. coli DH5α	Propagation of pET28a- and pJB3Tc20-derived plasmids	Thermo Fischer
P. aeruginosa CPHL12447	Indicator strain for PaeM ($PseuM_{NCTC10332}$)	J. P. Pirnay
P. aeruginosa NCTC10332	Strain encoding PaeM ($PseuM_{NCTC10332}$)	BCCM
P. fluorescens F113	Indicator strain for PflM ($PseuM_{Q8r1\text{-}96}$)	(Redondo-Nieto *et al.*, 2013)
P. fluorescens Q8r1-96	Strain encoding PflM ($PseuM_{Q8r1\text{-}96}$)	(Loper *et al.*, 2012)
P. syringae LMG 5456	Indicator strain for PsyM ($PseuM_{DC3000}$)	BCCM
P. syringae DC3000	Strain encoding PsyM ($PseuM_{DC3000}$)	(Buell *et al.*, 2003)

Supplementary Table S2. Primers used in this study.

Number	Sequence[a] (5' to 3')	Purpose of use
PGPRB-8461	GCTCACTCATTAGGCACCC	Sequencing of inserts in pJB3Tc20
PGPRB-9001	TGGCTACCATGGGTCCTATTGAGCTTCCTCCGACATACAT	Cloning of PseuM$_{DC3000}$ in pET28a (pCMPG6248)
PGPRB-9002	TGGCTACTCGAGGTTGCCACTAACCGTAACCGG	Cloning of PseuM$_{DC3000}$ in pET28a (pCMPG6248)
PGPRB-9003	TGGCTACCATGGGTGCAATGGATCTTGGTACCACAACAAT	Cloning of PseuM$_{NCTC10332}$ in pET28a (pCMPG6250)
PGPRB-9004	TGGCTACTCGAGACCAGAAATATTAACAGGGATAGTACCC	Cloning of PseuM$_{NCTC10332}$ in pET28a (pCMPG6250)
PGPRB-9005	TGGCTACCATGGGTGAATTCGAGCTTCCAGCTACTT	Cloning of PseuM$_{Q8r1-96}$ in pET28a (pCMPG6271)
PGPRB-9006	TGGCTACTCGAGTCGGGCGTAGCTAATAGGG	Cloning of PseuM$_{Q8r1-96}$ in pET28a (pCMPG6271)
PGPRB-9007	TGGCTACTGCAGCTTTCCATAAGGATTGACAATGGCTGC	Cloning of PmiA$_{DC3000}$ in pJB3Tc20 (pCMPG6251)
PGPRB-9008	TGGCTATCTAGAAGCGCCTGAAATCAGACGCTA	Cloning of PmiA$_{DC3000}$ in pJB3Tc20 (pCMPG6251)
PGPRB-9009	TGGCTACTGCAGACCATAAGAGGATGGTTTCATGAGA	Cloning of PmiA$_{NCTC10332}$ in pJB3Tc20 (pCMPG6252)
PGPRB-9010	TGGCTAGAATTCTTGTATCGCAGCTTTACGTCTACT	Cloning of PmiA$_{NCTC10332}$ in pJB3Tc20 (pCMPG6252)
PGPRB-9011	TGGCTACTGCAGAGGTTTTTGCAATAATATCGCTAGGAT	Cloning of PmiA$_{Q8r1-96}$ in pJB3Tc20 (pCMPG6269)
PGPRB-9012	TGGCTATCTAGACATCATCCTGCGCAGTCATG	Cloning of PmiA$_{Q8r1-96}$ in pJB3Tc20 (pCMPG6269)
PGPRB-9013	CTACAGCCTTCTGCTACCTATGGGCTG	Construction of a D36A mutation in PmiA$_{DC3000}$ (pCMPG6266)
PGPRB-9014	CAGCCCATAGGTAGCAGAAGGCTGTAG	Construction of a D36A mutation in PmiA$_{DC3000}$ (pCMPG6266)
PGPRB-9015	GTCAACCCCGATGCCACCACGGGTGTT	Construction of a D34A mutation in PmiA$_{NCTC10332}$ (pCMPG6267)
PGPRB-9016	AACACCCGTGGTGGCATCGGGGTTGAC	Construction of a D34A mutation in PmiA$_{NCTC10332}$ (pCMPG6267)
PGPRB-9017	AACCCAAGTGCTACATCTGGC	Construction of a D39A mutation in PmiA$_{Q8r1-96}$ (pCMPG6270)
PGPRB-9018	GCCAGATGTAGCACTTGGGTT	Construction of a D39A mutation in PmiA$_{Q8r1-96}$ (pCMPG6270)
PGPRB-10029	TGGCAGCAGCCAACTCAGCTT	Sequencing of inserts in pET28a
PGPRB-10030	TATAGGCGCCAGCAACCGCA	Sequencing of inserts in pET28a
PGPRB-10318	TGGCTACTGCAGCGATAGGCATAGAGAAGACATGAA	Cloning of PmiA$_{5}$ in pJB3Tc20 (pCMPG6272)
PGPRB-10319	TGGCTATCTAGAGCGGATGGATTGGTTGATT	Cloning of PmiA$_{5}$ in pJB3Tc20 (pCMPG6272)
PGPRB-10320	TGGCTACTGCAGTTTCTCTAGGTGAACAGCAATGGTAG	Cloning of PmiA$_{Pf153}$ in pJB3Tc20 (pCMPG6273)
PGPRB-10321	TGGCTATCTAGACCGCTCGTCCTGCAAAAGGC	Cloning of PmiA$_{Pf153}$ in pJB3Tc20 (pCMPG6273)
PGPRB-10322	TGGCTACTGCAGTTTTTCAAGGTAACCCACTATGATT	Cloning of PmiA$_{Pf29Arp}$ in pJB3Tc20 (pCMPG6274)
PGPRB-10323	TGGCTATCTAGATGCCCTGGGAAGGACAAG	Cloning of PmiA$_{Pf29Arp}$ in pJB3Tc20 (pCMPG6274)
PGPRB-10324	TGGCTACTGCAGTTTCTCTAGGTAAGTAGCCATGG	Cloning of PmiA$_{DF41}$ in pJB3Tc20 (pCMPG6275)

PGPRB-10325	TGGCTATCTAGAAAGAGTAATCTTCACCGCGCA	Cloning of $PmiA_{DF41}$ in pJB3Tc20 (pCMPG6275)
PGPRB-10326	TGGCTACTGCAGATAACAGGAGCAACACTCCATG	Cloning of $PmiA_{GM21}$ in pJB3Tc20 (pCMPG6276)
PGPRB-10327	TGGCTATCTAGAGCCTAAAGATGTTCAAGGGAGT	Cloning of $PmiA_{GM21}$ in pJB3Tc20 (pCMPG6276)
PGPRB-10328	TGGCTACTGCAGAAGAGCGTGGAATATTTAAATGAATCT	Cloning of $PmiA_{AU2390}$ in pJB3Tc20 (pCMPG6277)
PGPRB-10329	TGGCTATCTAGAAAACTCATGATCGAGCGTACT	Cloning of $PmiA_{AU2390}$ in pJB3Tc20 (pCMPG6277)

[a] Restriction sites incorporated in the primers are underlined: GAATTC, EcoRI; CCATGG, NcoI; CTGCAG, PstI; TCTAGA, XbaI;CTCGAG, XhoI.

Figure S1. (A) Multiple sequence alignment of ColM domains derived from unique PseuM proteins from *Pseudomonas* spp. Abbreviations for species names are as in Figure 1. Other abbreviations: Pced, *Pseudomonas cedrina*; Pcor ory, *Pseudomonas coronafaciens* pv. *oryzae*; Pden, *Pseudomonas denitrificans*; Pkor, *Pseudomonas koreensis*; Ppro, *Pseudomonas protegens*; Psyr ace-cori-mors-syr-the, *Pseudomonas syringae aceris-coriandricola-morsprunorum-syringae-theae*; Pthi, *Pseudomonas thivervalensis*; Pver, *Pseudomonas veronii*. Grey shading reflects the degree of conservation. (B) Unrooted maximum likelihood phylogenetic tree of ColM domains of pseudomonads (black) from panel (A) and previously characterized ColM domain-carrying bacteriocins from other β- and γ-proteobacteria: colicin M (ColM, red) from *E. coli*, pectocin M1 and M2 from *Pectobacterium* spp. (PecM1/PecM2, pink), burkhocin M1 and M2 from *Burkholderia* spp. (BurM1/BurM2, grey). *Pseudomonas* abbreviations as in (A). The clade of *Pseudomonas* ColM domains from bacteriocins linked with a *pmiA*-type immunity gene and *pmiB*-type immunity gene are in a red arc and a blue ellipse, respectively. Other abbreviations: Bamb, *Burkholderia ambifaria*; Ecoli, *Escherichia coli*; Pcar, *Pectobacterium carotovorum*. The scale represents 0.6 substitutions per site. Bootstrap values (percentages of 1000 replicates) are not shown for visibility.

A

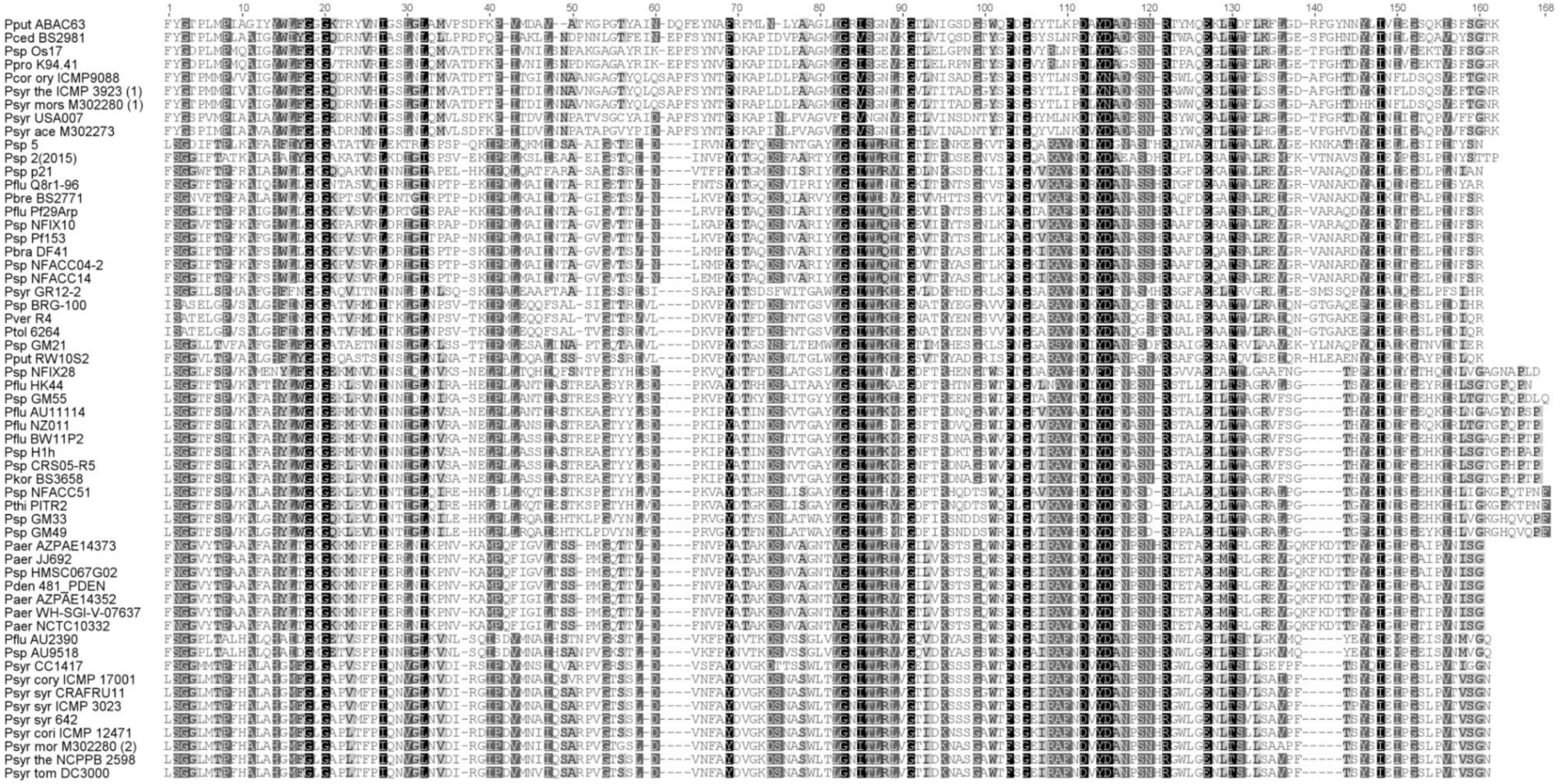

Pput ABAC63
Pced BS2981
Psp Os17
Ppro K94.41
Pcor ory ICMP9088
Psyr the ICMP 3923 (1)
Psyr mors M302280 (1)
Psyr USA007
Psyr ace M302273
Psp 5
Psp 2(2015)
Psp p21
Pflu Q8r1-96
Pbre BS2771
Pflu Pf29Arp
Psp NFIX10
Psp Pf153
Pbra DF41
Psp NFACC04-2
Psp NFACC14
Psyr GR12-2
Psp BRG-100
Pver R4
Ptol 6264
Psp GM21
Pput RW10S2
Psp NFIX28
Pflu HK44
Psp GM55
Pflu AU11114
Pflu NZ011
Pflu BW11P2
Psp H1h
Psp CRS05-R5
Pkor BS3658
Psp NFACC51
Pthi PITR2
Psp GM33
Psp GM49
Paer AZPAE14373
Paer JJ692
Psp HMSC067G02
Pden 481_PDEN
Paer AZPAE14352
Paer WH-SGI-V-07637
Paer NCTC10332
Pflu AU2390
Psp AU9518
Psyr CC1417
Psyr cory ICMP 17001
Psyr syr CRAFRU11
Psyr syr ICMP 3023
Psyr syr 642
Psyr cori ICMP 12471
Psyr mor M302280 (2)
Psyr the NCPPB 2598
Psyr tom DC3000

B

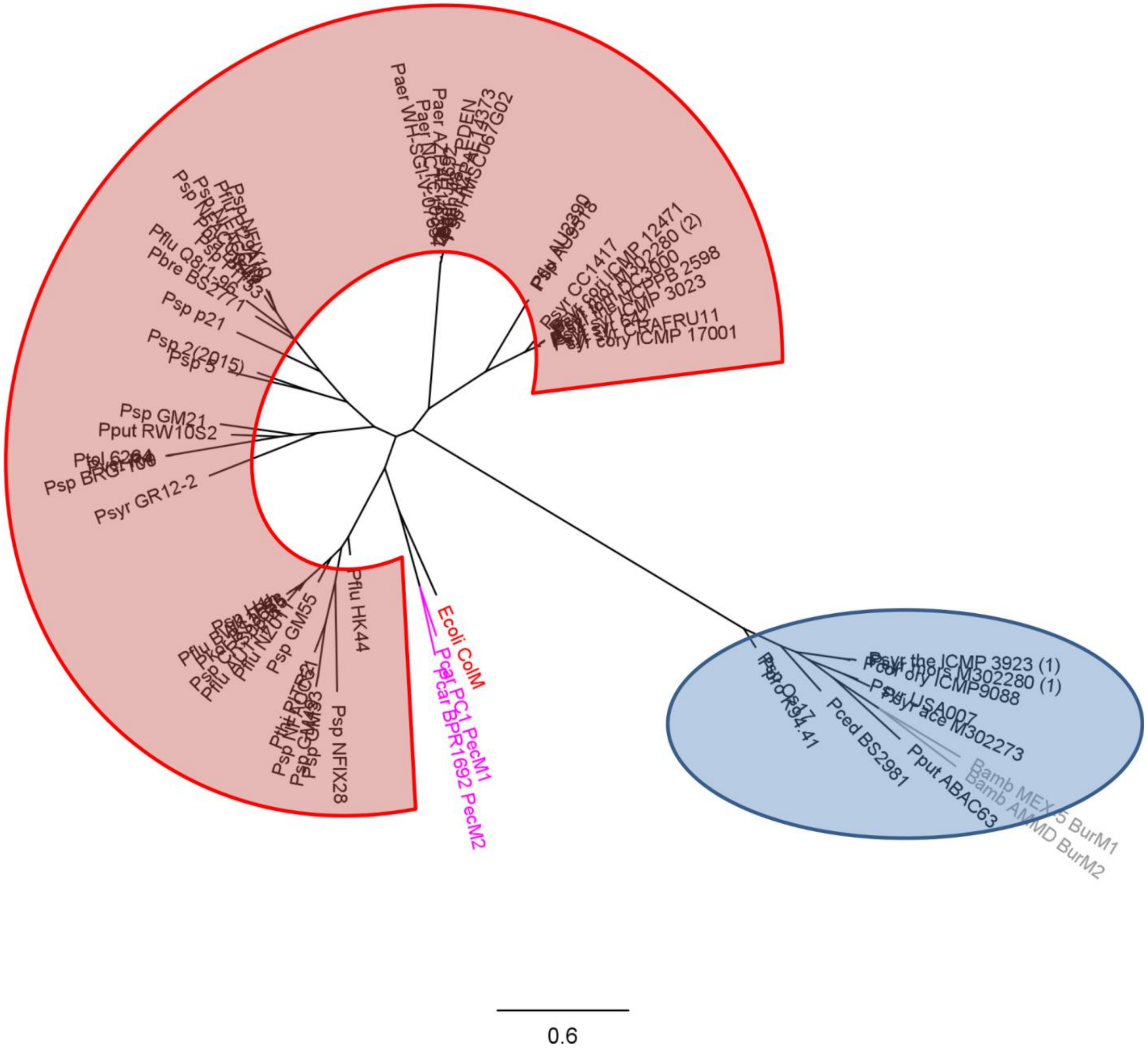
Psp p21
Pbre BS2771
Psp 2(2015)
Psp 5
Psp GM21
Pput RW10S2
Psp BRG100
Psyr GR12-2
Pflu HK44
Psp GM55
Psp NFIX28
Ecoli ColM
Pcar PC1 PecM1
Pcar BPR1692 PecM2
Psyr CC1417
Psyr cory ICMP 17001
Psyr the ICMP 3923 (1)
Psyr USA007
Psyr ace M302273
Psp Os17
Pced BS2981
Pput ABAC63
Bamb MEX-5 BurM1
Bamb AMMD BurM2
0.6

Figure S2. Multiple sequence alignment of BmiB (*Burkholderia* spp.) and unique PmiB (*Pseudomonas* spp.) proteins. Grey shading indicates the degree of conservation. Abbreviations for *Burkholderia* species names: Bubo, *Burkholderia ubonensis*; Bcon, *Burkholderia contaminans*; Bamb, *Burkholderia ambifaria*; Bokl, *Burkholderia oklahomensis*; Bcep, *Burkholderia cepacia*; Bcen, *Burkholderia cenocepacia*; Bpyr, *Burkholderia pyrrocinia*; Burk, *Burkholderia* sp. *Pseudomonas* abbreviations are as in Figure 1 and Figure S1. Predicted transmembrane helices are boxed in blue, Sec- or Tat-dependent signal sequences in red and lipoprotein signal sequences in yellow. Two conserved cysteine residues are highlighted in green.

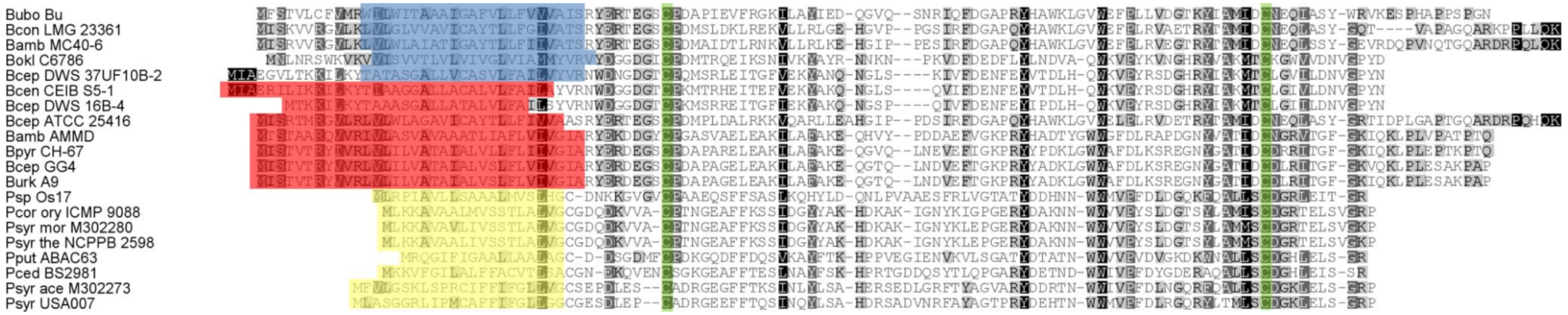

Figure S3. Multiple sequence alignment of unique PmiA proteins. Predicted transmembrane helices are boxed in blue, Sec- or Tat-dependent signal sequences in red. Grey shading indicates the degree of conservation. *Pseudomonas* species abbreviations are as in Figure S1. This multiple sequence alignment was used for construction of phylogenetic tree in Figure 1B.

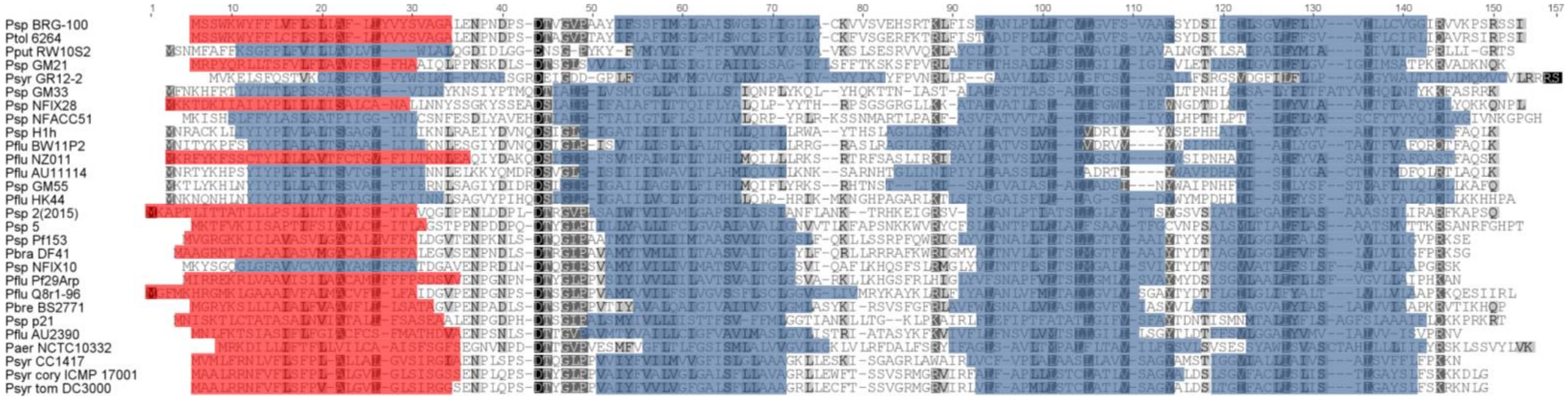

References

Blatny, J. M., Brautaset, T., Winther-Larsen, H. C., Haugan, K. and Valla, S. (1997). Construction and use of a versatile set of broad-host-range cloning and expression vectors based on the RK2 replicon. *Appl. Environ. Microbiol.* 63, 370-379.

Buell, C. R., Joardar, V., Lindeberg, M., Selengut, J., Paulsen, I. T., Gwinn, M. L., et al. (2003). The complete genome sequence of the *Arabidopsis* and tomato pathogen *Pseudomonas syringae* pv. *tomato* DC3000. *Proc. Natl. Acad. Sci. U. S. A.* 100, 10181-10186.

Loper, J. E., Hassan, K. A., Mavrodi, D. V., Davis, E. W., 2nd, Lim, C. K., Shaffer, B. T., et al. (2012). Comparative genomics of plant-associated *Pseudomonas* spp.: insights into diversity and inheritance of traits involved in multitrophic interactions. *PLoS Genet.* 8, e1002784. doi: 10.1371/journal.pgen.1002784

Redondo-Nieto, M., Barret, M., Morrissey, J., Germaine, K., Martinez-Granero, F., Barahona, E., et al. (2013). Genome sequence reveals that *Pseudomonas fluorescens* F113 possesses a large and diverse array of systems for rhizosphere function and host interaction. *BMC Genomics* 14, 54. doi: 10.1186/1471-2164-14-54
